# The impact of meteorological factors on tuberculosis incidence in Spain: a spatiotemporal analysis

**DOI:** 10.1017/S0950268824000499

**Published:** 2024-03-20

**Authors:** María del Mar Díez Galán, Lidia Redondo-Bravo, Diana Gómez-Barroso, Laura Herrera, Rocio Amillategui, Javier Gómez-Castellá, Zaida Herrador, Spanish Working Group on Tuberculosis

**Affiliations:** 1 Preventive Medicine Department Hospital General Universitario de Elche, Alicante, Spain; 2 Health Emergencies Department, Pan American Health Organization, Washington, DC, USA; 3 National Center of Epidemiology, Instituto de Salud Carlos III, Madrid, Spain; 4 CIBER in Epidemiology and Public Health (CIBERESP), Madrid, Spain; 5 Department of Bacteriology, National Centre of Microbiology, Instituto de Salud Carlos III, Majadahonda, Spain; 6 División de control de VIH, ITS, Hepatitis virales y Tuberculosis. Ministerio de Sanidad, Madrid, Spain

**Keywords:** geospatial analysis, solar radiation, Spain, tuberculosis, Vitamin D, Time series

## Abstract

Tuberculosis (TB) remains a global leading cause of death, necessitating an investigation into its unequal distribution. Sun exposure, linked to vitamin D (VD) synthesis, has been proposed as a protective factor. This study aimed to analyse TB rates in Spain over time and space and explore their relationship with sunlight exposure. An ecological study examined the associations between rainfall, sunshine hours, and TB incidence in Spain. Data from the National Epidemiological Surveillance Network (RENAVE in Spanish) and the Spanish Meteorological Agency (AEMET in Spanish) from 2012 to 2020 were utilized. Correlation and spatial regression analyses were conducted. Between 2012 and 2020, 43,419 non-imported TB cases were reported. A geographic pattern (north–south) and distinct seasonality (spring peaks and autumn troughs) were observed. Sunshine hours and rainfall displayed a strong negative correlation. Spatial regression and seasonal models identified a negative correlation between TB incidence and sunshine hours, with a four-month lag. A clear spatiotemporal association between TB incidence and sunshine hours emerged in Spain from 2012 to 2020. VD levels likely mediate this relationship, being influenced by sunlight exposure and TB development. Further research is warranted to elucidate the causal pathway and inform public health strategies for improved TB control.

## Key results

1. Tuberculosis (TB) rates in Spain displayed a distinct north–south geographic pattern. This geographical variation highlights the need for tailored interventions, directing resources and efforts to regions with a higher TB incidence.
2. The study identified clear seasonality in TB rates, with peaks in spring and troughs in autumn. Recognizing these seasonal trends enables healthcare systems and public health authorities to prepare for increased TB cases during peak seasons and adjust strategies accordingly.
3. Sunshine hours showed a strong negative correlation with TB incidence. This suggests that increased sunlight exposure may have a protective effect against TB, emphasizing the potential role of sun exposure in reducing disease risk.
4. Vitamin D (VD) levels likely mediate the association between sunlight exposure and TB incidence. Understanding this link provides insights into the importance of adequate VD levels and suggests potential avenues for preventive measures in TB control.

## Introduction

Tuberculosis (TB) is still one of the leading causes of death in the world. European Union and European Economic Area (EU/EEA) countries reported 33,148 TB cases in 2020 (notification rate 7.3 per 100,000 population), which accounted for 2% of the global burden [1, 2]. During the period 2011–2020, the average annual decrease in TB incidence in the EU/EEA was 5.2% (6.4% between 2019 and 2020) [3]. In Spain, between 2015 and 2020, the incidence declined on average by 5.4% per year [4]. Considering the remarkable progress achieved in the last decade, the main challenge in low-incidence countries such as Spain (notification rate below 10 per 100,000 population) is to continue moving towards elimination [2, 5, 6]. In order to guide the implementation of control measures, identifying clusters as well as vulnerable populations and potential risk factors is currently challenging. In Spain, the distribution of TB incidence rates is heterogeneous among the different autonomous communities (CCAA in Spanish), with higher incidence rates detected in the northern regions and in the autonomous cities of Ceuta and Melilla [4].

Traditional TB risk factors are medical conditions or underlying diseases that cause immunosuppression. Lifestyle habits such as smoking and alcoholism and social determinants such as poverty or overcrowding are also determinants. However, these factors may not completely explain the variations in TB incidence in low-incidence countries [7]. Vitamin D (VD) levels have been identified as another possible factor associated with TB progression. VD is derived primarily from cutaneous synthesis through solar exposure to ultraviolet B (UVB) radiation and, to a lesser extent, from diet [7, 8]. The biologically active form, 1,25-dihydroxyvitamin D, reduces intracellular growth of *Mycobacterium tuberculosis* through modulation of macrophage function. Adequate levels of VD are associated with increased innate immune response and reduced initial bacterial invasion [8]. Moreover, several studies suggest that VD deficiency may be associated with increased TB incidence, severity, or progression [7–10].

On the other hand, sun exposure has been identified as a possible protective factor for TB. This protection has been associated with the synthesis of VD in the skin after UVB radiation exposure [7, 11]. This implies that the less a person is exposed to sunlight, the greater the risk of developing TB [12].

In addition, meteorological factors are thought to influence the incidence of TB by affecting to some extent the growth and reproduction of *M. tuberculosis.* While long exposure to sunlight may limit the development of *M. tuberculosis*, as it is very sensitive to ultraviolet light, increased rainfall may create a suitable environment for its growth and reproduction [13–15].

To better understand the epidemiology of TB in relation to these environmental factors in Spain, we aimed to study the spatial and temporal patterns of TB rates and to identify whether there is a relationship between solar radiation and rainfall and its incidence between 2012 and 2020.

## Methods

### Study design

Based on TB cases reported to the National Epidemiological Surveillance Network (RENAVE in Spanish) and data from the Spanish Meteorology State Agency (AEMET in Spanish), we performed a retrospective spatiotemporal ecological study to assess the association between potential environmental risk factors (rainfall and sunshine hours) and the incidence rates of TB in Spain, for total cases and by disease main location.

#### Data sources

Autochthonous TB cases reported to the RENAVE by the Spanish Autonomous Regions between 2012 and 2020 were included in the study. Suspected, probable, and confirmed TB cases are reported on a weekly basis by the surveillance units from the CCAA in a case-based format through the Spanish Surveillance System (SiViEs in Spanish). By using the case report form, the CCAA updates the information initially reported, and a consolidation process is performed yearly [5].

The following clinical and sociodemographic variables from the RENAVE database were extracted: date of birth, date of symptoms’ onset, age, sex, province, CCAA, and TB location. Province and CCAA refer to the place where cases got infected.

The TB location was used to classify the cases into pulmonary or extrapulmonary forms. According to the definitions provided in the RENAVE protocol, pulmonary TB is that affecting lung parenchyma and the tracheobronchial tree. Extrapulmonary TB was considered when any other location was affected (pleural, lymphatic, osteoarticular, central nervous system, genitourinary, digestive/peritoneal, disseminated, and other extrapulmonary locations) [5].

The official population from the National Statistics Institute (INE in Spanish) [14] was used as the ‘population at risk’ for the study period.

Environmental variables were obtained from the AEMET open database (available at Base de datos histórica de Precipitaciones y horas de sol. (datosclima.es)).

Daily values from the main meteorological station in each province from 2012 to 2020 were used. The monthly sunshine hours are shown as the average number of sunshine hours per month. The unit of measurement for the average rainfall per month was litres/m^2^ (l/m^2^). Negligible precipitation values were replaced by 0 for the calculations.

#### Ethical statement

This study involves the use of epidemiological data from the RENAVE. These data are hosted by the National Center of Epidemiology (CNE). Researchers working in public and private institutions can request the databases by completing, signing, and sending a questionnaire. In this questionnaire, a signed confidentiality commitment is required. According to this confidentiality commitment, researchers cannot provide the data to other researchers, who must request the data directly from the CNE. All data are anonymized and de-identified. Data from the National Statistics Institute (INE) and the AEMET were obtained through open data access. Thus, no ethical approval or informed consent was required by the Spanish Human Research Act to conduct this data analysis.

## Data analysis

### Description

A description of the annual TB incidence rate trends, the environmental factors, and the basic demographic characteristics of the TB cases was performed.

The incidence rates by age group and sex were adjusted by applying the direct method, being categorized into 13 age groups.

Central tendency and dispersion measures (median (Mdn) and interquartile range (IQR)) were used for quantitative variables. Qualitative variables were expressed as absolute and relative frequencies (%).

The average incidence TB rates by 100,000 inhabitants in all countries and by province were calculated for the period 2012–2020, by year and month, considering the total cases as well as the pulmonary and extrapulmonary forms. Incidence rates for total, pulmonary, and extrapulmonary TB by province were mapped to describe the geographical distribution during the studied period.

The national average and the average by province were calculated for environmental variables. Mean (M) and standard deviation (SD) were used for sunshine hours and rainfall. The average sunshine hours and average rainfall were mapped to identify their geographic distribution in the different provinces of Spain.

### Spatial regression analysis

Moran’s spatial correlation index (Moran’s I) was used to analyse the global autocorrelation (spatial component) at the province level of total, pulmonary, and extrapulmonary TB rates.

The correlation between sunshine hours and rainfall was assessed by the Pearson correlation coefficient. A p-value of <0.05 was considered statistically significant.

A linear regression analysis was performed. The residuals were analysed using Moran’s I to study whether there was a spatial component [16]. Once the autocorrelation was detected, a regression model with spatial lag was performed to assess the relationship between the total, pulmonary, and extrapulmonary TB incidence rates with environmental variables.

### Time-series and cross-correlation analyses

To assess the trend in the TB monthly rates, a linear regression was performed. Additionally, to explore the seasonal pattern in the time series, a detrend by fitting a linear regression model to the data was performed, and then, the difference between the observed values and the predicted values from the model was calculated.

To model the seasonality of the TB monthly rates (model A) and the average monthly sunshine hours (model B), a regression analysis was performed including the previously mentioned variables as dependent variables (each one in the respective model A or B) and seasonality as independent variable by using the sine and cosine functions.

A cross-correlation analysis was performed to assess the correlation and the potential lags between the TB monthly rates (model A and unmodelled values) and the average monthly daylight hours (model B and unmodelled values).

#### Statistical software

All the data were included in Microsoft Excel® and analysed with the IBM Statistical Package for the Social Sciences (SPSS) Statistics version 28, and the statistical software for data science (Stata) v17.0 GeoDa was used for spatial analysis and Quantum Geographic Information System (QGIS) v3.18 for map representation.

## Results

### Description of the study population

Between 2012 and 2020, 44,838 TB cases were reported to the RENAVE, 1,419 of which were imported cases and therefore excluded. The remaining 43,419 cases were finally included.

**Figure 1.**
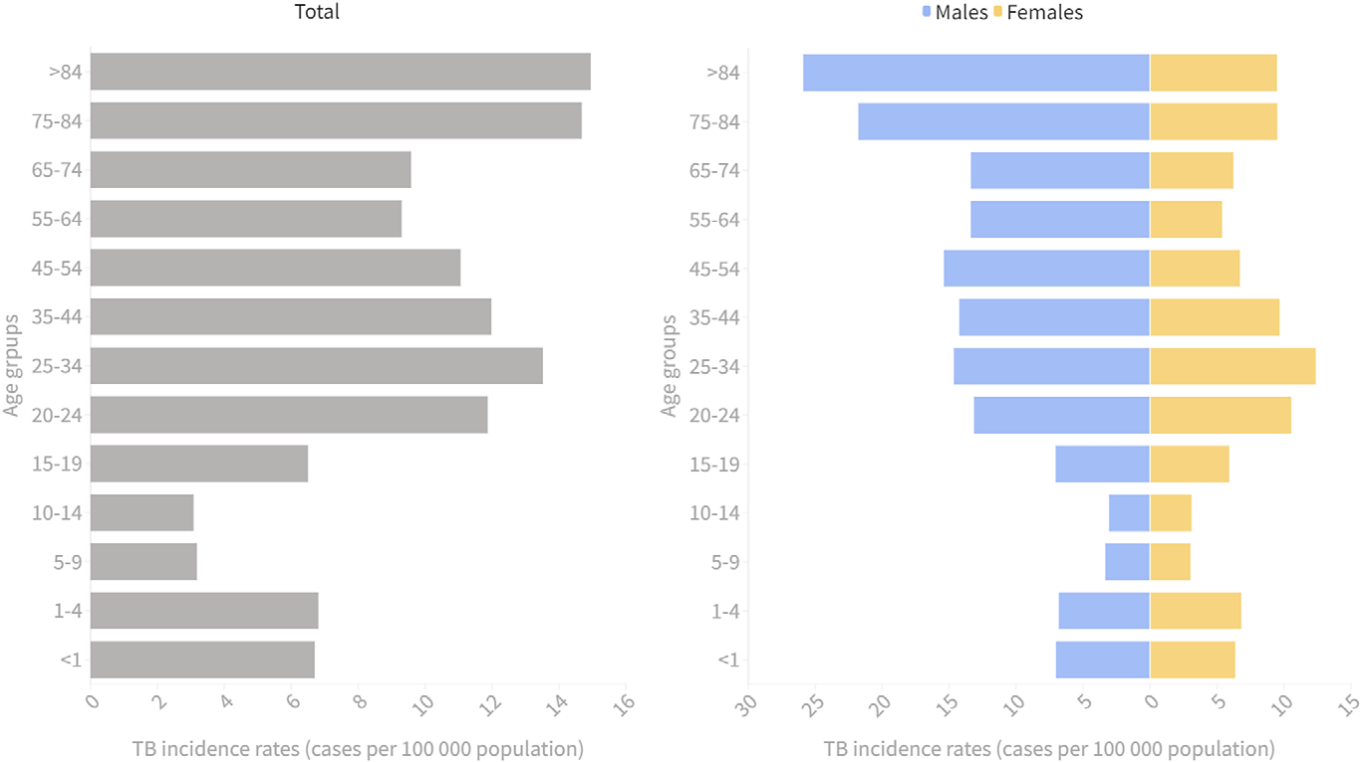
Adjusted incidence rates of TB per 100,000 inhabitants by age groups and sex, Spain 2012-2020

TB was more frequent among males in all age groups, and these differences tend to increase with age. There is a men-to-women ratio of 1.6 (Figure 1).

The overall Mdn age was 44 years (IQR = 31–61). The Mdn age was significantly higher among males (Mdn = 46, IQR = 33–62) than females (Mdn = 41, IQR = 28–60).

Considering the distribution of TB cases according to location, 70% (n = 30,446) of the cases presented pulmonary TB and 25% (n = 11,136) of the cases presented extrapulmonary TB, and this information was missing in 5% of the records (1,837 cases). By sex, pulmonary TB was more frequent in men, who accounted for 64.8% (19,726) of cases compared to women (p < 0.05). Although statistically significant (p < 0.05), a small difference was observed by sex in the extrapulmonary forms (54.1% (6,021) in males and 45.9% (5,115) in females).

**Figure 2.**
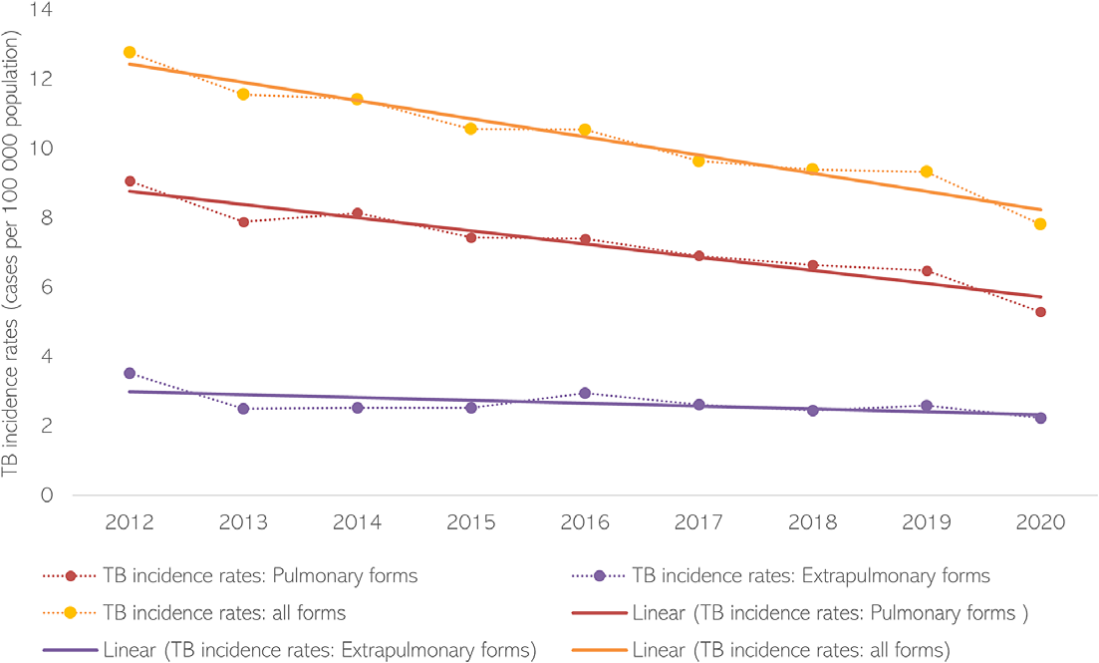
Annual average of TB incidence rate in Spain, 2012-2020

The average TB incidence rate throughout the studied period was 10.33 per 100,000 inhabitants for all TB forms, 7.24 per 10^5^ inhabitants for pulmonary TB, and 2.65 per 10^5^ inhabitants for extrapulmonary TB. Annual incidence rates showed a decreasing trend for all TB forms (regression coefficient − 1.8; 95% confidence interval (CI): −2.2 to −1.4). When performing the linear regression by TB type, only pulmonary TB showed a significant decreasing trend (regression coefficient − 2.4, 95% CI: −3.1 to −1.8 for pulmonary vs. -4.4, 95% CI: −9.6 to 0.8 for extrapulmonary forms, respectively) (Figure 2).

**Figure 3.**
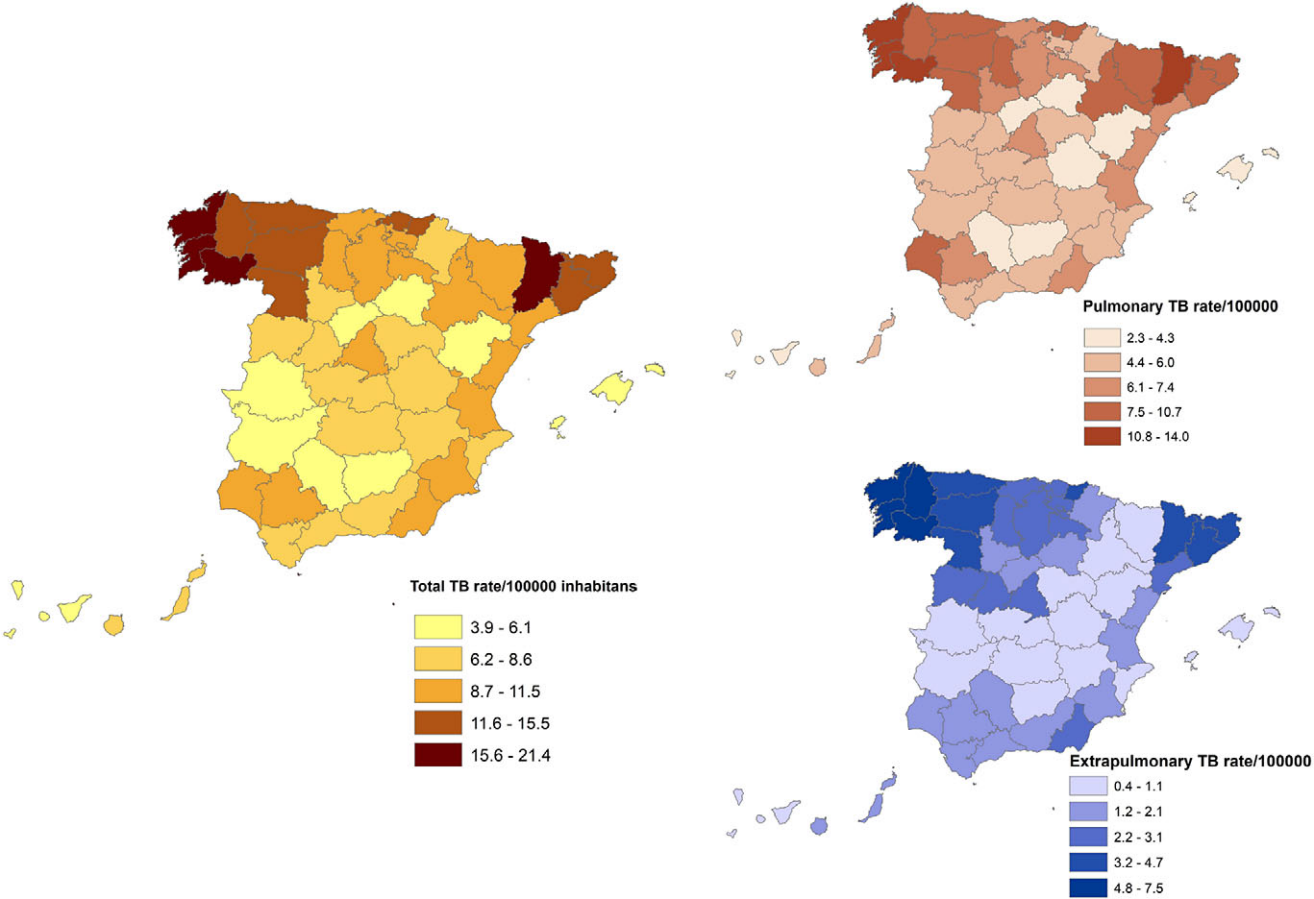
Distribution of TB rates per 100,000 inhabitants by location and province in Spain, 2012-2020

The provinces with higher average incidence rates per 100,000 inhabitants throughout the studied period were A Coruña (21.3), Pontevedra (20.7), Ourense (19.8), Ceuta (18.4), Lleida (18.3), Melilla (17.0), León (15.4), Lugo (15.0), Gipuzkoa (14.0), and Barcelona (14.0), all of them above the national average (10.3). The geographical distribution of TB rates is similar for both forms, with a north–south gradient (higher incidence rates in the north than in the south). Figure 3 shows the incidence rate maps by province and TB location. These incidence rates were calculated including the cases where the province was specified (n = 42.888, 98.8%).

#### Description of environmental variables

The sunshine hours and rainfall remained relatively steady throughout the 2012–2020 period, showing seasonal cycles. For sunshine hours, peaks were observed in the summer (July), while troughs were detected in late autumn–early winter (November–January). For rainfall, peaks were observed during late autumn–early winter (November–January) and troughs during the summer (July–August) (Supplementary Figure S1).

At the national level, there was an average of 7.52 h of sunshine (SD 0.25) with a minimum of 7.25 in 2018 and a maximum of 8.01 in 2017. Sevilla, Huelva, Alicante, Almeria, and Murcia were the provinces with the highest values, with an average of 9.20, 9.18, 9.00, 8.88, and 8.87, respectively. The average rainfall was 1.52 l/m2 (SD 0.22), with a minimum of 1.20 in 2017 and a maximum of 1.93 in 2018. The provinces with the highest levels of rainfall were Gipuzkoa, Pontevedra, Bizkaia, Asturias, and Cantabria, with an average of 5.19, 4.19, 3.42, 3.11, and 3.08 l/m^2^, respectively.

**Figure 4.**
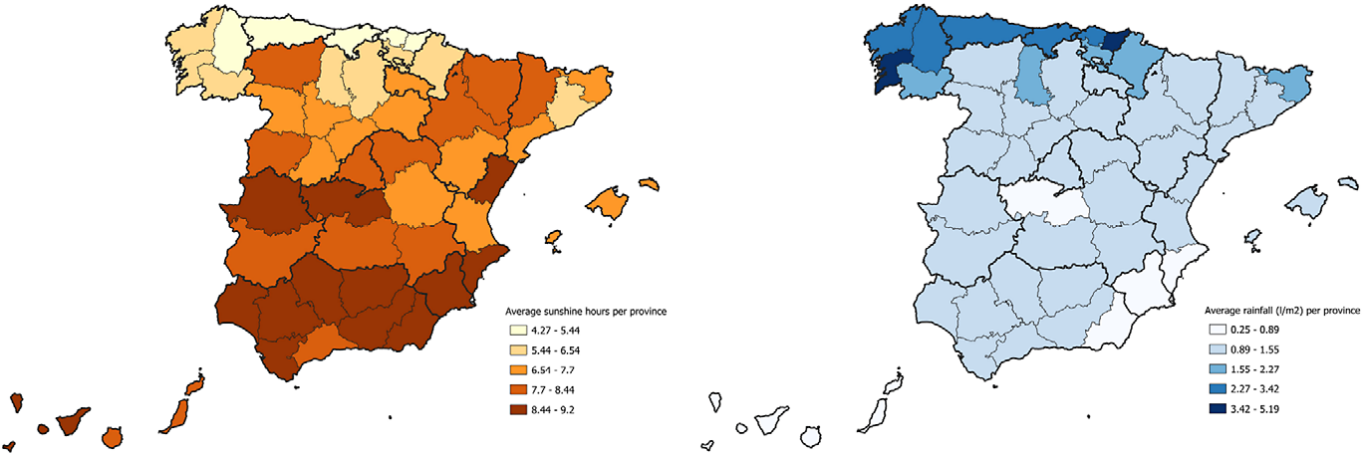
Distribution map of average rainfall and sunshine hours by provinces in Spain in the period 2012-2020

A south–north gradient was observed, with a greater number of sunshine hours in the southern provinces of Spain, compared to those in the north, and a north–south gradient in rainfall, with higher values in the northern provinces compared to the southern ones (Figure 4).

#### Spatial regression analysis

Moran’s indexes were 0.67 for the total TB rate, 0.57 for the pulmonary TB rate, and 0.75 for the extrapulmonary TB rate. This indicates a high spatial autocorrelation between TB rates at the province level.

Pearson’s correlation coefficient between the average sunshine hours and average rainfall was −0.81 (*p* < 0.01), proving a strong negative correlation between both variables. To avoid collinearity, only sunshine hours was included in the final models.

After performing basic regression analyses to assess the spatial autocorrelation among the residuals (Moran’s I >0.4), three spatial regression models with spatial lag were performed to assess the association between total, pulmonary, and extrapulmonary TB rates with mean sunshine hours. The total TB and extrapulmonary TB models were significant, while the pulmonary TB model was not significant (Table 1).

**Table 1.** Spatial lag model for total, pulmonary, and extrapulmonary TB rates and average sunshine hours in Spain, 2012–2020

Spatial lag model (TB and average sunshine hours)	*R*-square	Coefficient	*p*-value
Total TB	0.31	−1.03	0.02
Pulmonary TB	0.18	−0.57	0.07
Extrapulmonary TB	0.58	−0.3	0.02

#### Time series and cross-correlation

**Figure 5.**
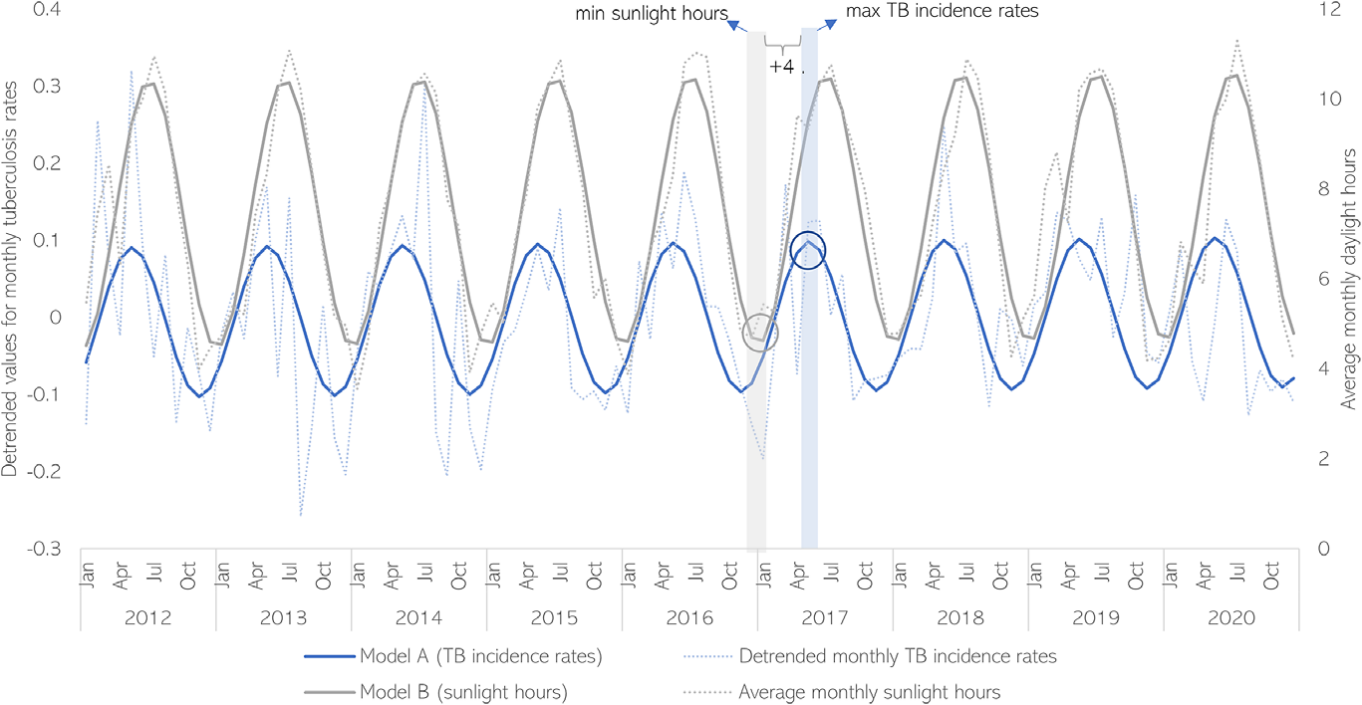
Monthly tuberculosis rates (raw and model A) and the average monthly daylight hours (raw and model B), Spain, 2012-2020

The detrended monthly TB rates (raw and model A) and the average monthly daylight hours (raw and model B) are plotted in Figure 5.

A strong negative correlation was detected in lag −4 (cross-correlation coefficient = −0.94) in the correlation analysis between model A and model B, which means that there is a strong negative correlation between the modelled value for the monthly TB rates in month 0 and the modelled value for the average monthly sunshine hours four months earlier (month 4). A strong positive correlation with the average monthly daylight hours in month +1 was also found.

When performing the cross-correlation analysis with the (non-modelled) detrended TB monthly rates and the average monthly daylight hours, the lags of maximum correlation were also −4 (correlation coefficient = −0.51) and + 1 (correlation coefficient = 0.55).

According to the modelled cycles, peaks of TB incidence were observed in spring (May) 4 months after the trough of sunlight observed in winter (January) (lag of 4 months), while the trough of TB incidence usually occurs in autumn (November), 4 months after the peak in sunlight observed during summer (July) (lag of 4 months).

## Discussion

To the best of our knowledge, this is the first study providing spatiotemporal patterns for TB incidence rates and assessing its association with environmental factors to address the heterogeneous distribution of TB in Spain. In the light of our results, a negative association between TB incidence rates and mean sunshine hours exists, both at geographical and temporal levels.

The distribution of TB cases by sex was similar to what has been described globally and in other European series, with higher rates among men than women [17–19]. By age groups, the highest incidence rates were found among those of 25–34 and 75+ years old; however, when stratifying by sex, the highest incidence rates in men of 75+ years old and women between 25 and 34 years old were observed. These sex and age differences have been previously described, pointing out immunological reasons leading to a different response to infection at different ages. Behavioural risk factors, usually more frequent in men than in women (i.e. imprisonment, alcohol abuse, drug use, or smoking), seem to also play an important role [17, 20].

When analysing the temporal evolution of TB incidence rates, we observed a significant decreasing trend in all forms of TB and the pulmonary forms, but extrapulmonary forms, which are more common in immunosuppressed population (including human immunodeficiency virus (HIV)+) and children, remained steadier. These results agree with the data reported by the World Health Organization (WHO) European Region – European Centre for Disease Prevention and Control (ECDC) [18]. Extrapulmonary forms have been typically underreported due to their challenging diagnosis, which can lead to a late diagnosis and treatment. For these reasons, patients with extrapulmonary TB have been often neglected in TB control strategies. Additionally, the improvements in diagnosis and reporting in recent years may have influenced the absence of the decreasing trend due to an increase in the reporting of these forms. On the other hand, the geographical distribution of TB rates, with a north–south gradient – higher incidence rates in the north and lower incidence rates in the south – showed a similar pattern for both pulmonary and extrapulmonary forms.

In the spatial regression analysis, a negative correlation between the TB incidence and the sunshine hours was shown. The association between geographical patterns of TB and environmental factors, including sunshine hours, has also been observed in other studies in America and Asia with North–South (Chile) and West–East (China) patterns [7, 13, 14], related to solar radiation, rainfall, and humidity. While transmission is more likely to occur in enclosed spaces, influenced by cold and rainy weather, it is important to consider the role of the immune response in this association. Moreover, since TB is a latent infection, it is possible that the location where the case is identified may not necessarily reflect the location of the initial infection.

Supporting these findings, the time-series analysis also showed a strong negative correlation between the modelled TB incidence rate and the modelled sunshine hours with a lag of −4 months, meaning that after 4 months of low exposure to sunlight, peaks of TB incidence are observed. This reflects that the peaks in TB incidence rates observed around May in Spain are strongly correlated with the low sunlight hours observed during the previous winter (especially January). Several studies in different countries across the globe have also found a negative association between TB incidence and sunlight and a positive association between rainfall and TB incidence [21–23]; however, the findings regarding the lags differ, probably due to several environmental and non-environmental factors such as seasonal patterns and the presence of other risk factors that promote the development of the disease. In our study, we detected a strong negative correlation between sunshine hours and rainfall, so to evaluate its association with TB incidence we only performed the analysis with sunshine hours. We must consider that TB is a latent infection that may be triggered by several aspects closely related to immune response, and for this reason, a unique explanation for these ecological findings is hard to provide [24]. Nevertheless, the effect of sunlight on VD has been proposed as one feasible explanation [9, 23]. The mechanisms behind this are probably multifactorial including the effect of ultraviolet light on VD synthesis and its role in the immune response against TB [10, 25], as well as the fact that abundant rainfall could also create an environment suitable for the growth and reproduction of *M. tuberculosis,* while a longer duration of sunshine would restrict its development [26]. Other explanations for seasonality as healthcare activity seem to be unlikely since the peak coincides with the summer months, contradicting the theory of lower healthcare capacity due to summer vacations. The fact that the spatial correlation observed in Spain was stronger in the extrapulmonary forms, which are more common among immunocompromised individuals, may suggest that the effect of the sunshine hours on the incidence rates of TB may be highly mediated by the immune response. These findings highlight the importance of assessing the impact of detecting and treating VD deficiency on TB incidence in Spain, especially after months of the lowest sunlight, and on vulnerable populations. Nevertheless, this must be carried out together with other public health interventions oriented to early detection and treatment of cases and identification of close contacts for chemoprophylaxis, which are probably the leading causes of the TB decreasing trend observed during this period.

Our study presents some limitations associated with the use of reported cases: underreporting, missing information, or lack of information on common medical conditions and other TB risk factors. Nevertheless, the fact that most findings in this study are consistent with current literature provides an insight into how solid the surveillance data included in this study are. We may remain vigilant, and epidemiological surveillance systems should be strengthened to improve survey completion and avoid differences in reporting among regions, and other approaches including latent TB in surveillance and control strategies may be considered. In order to obtain reliable information for public health decision-making, the quality of this information should improve, increasing the accuracy of the data related to the different factors involved in the incidence of TB in Spain.

## Conclusions

During 2012–2020, a geographical pattern (with a north–south gradient) and a clear seasonality (peaks in spring and troughs in autumn) were observed. These patterns have shown a correlation with sunlight hours, which has been related to VD synthesis in other studies, highlighting the potential importance of this approach for the TB control strategy. Additionally, by using spatiotemporal methods, disease distribution patterns are well defined identifying high-risk regions in the north of Spain. These methods may be considered as complementary tools for the epidemiological surveillance of infectious diseases, which could provide important information to guide public health interventions and resource allocation. The reinforcement of public health measures considering all these aspects and the enhancement of epidemiological surveillance continue to be cornerstones towards TB elimination.

## Supporting information

Díez Galán et al. supplementary material Díez Galán et al. supplementary material

## Data Availability

The environmental data that support the findings of this study are openly available in the AEMET open database, which can be accessed at Base de datos histórica de Precipitaciones y horas de sol. (datosclima.es). The surveillance data used in this study can be made available upon request to the Spanish Surveillance System (SiViEs in Spanish). Researchers interested in accessing the data are invited to submit an official request form to sivies@isciii.es, providing a detailed rationale for their request. All requests will be subject to review by the data custodian and will be considered in accordance with relevant data protection and privacy regulations. Official population data from the National Statistics Institute (INE in Spanish) can be found at https://www.ine.es/dyngs/INEbase/es/operacion.htm?c=Estadistica_C&cid=1254736177095&menu=ultiDatos&idp=1254735572981.

## Supplementary material

For supplementary material accompanying this paper visit http://doi.org/10.1017/S0950268824000499. click here to view supplementary material
