# Supplementary figures and images for: The impact of meteorological factors on tuberculosis incidence in Spain: a spatiotemporal analysis
Source: Epidemiol Infect. 2024 Mar 20;152:e58. doi: 10.1017/S0950268824000499 (PMC11022253; doi:10.1017/S0950268824000499)

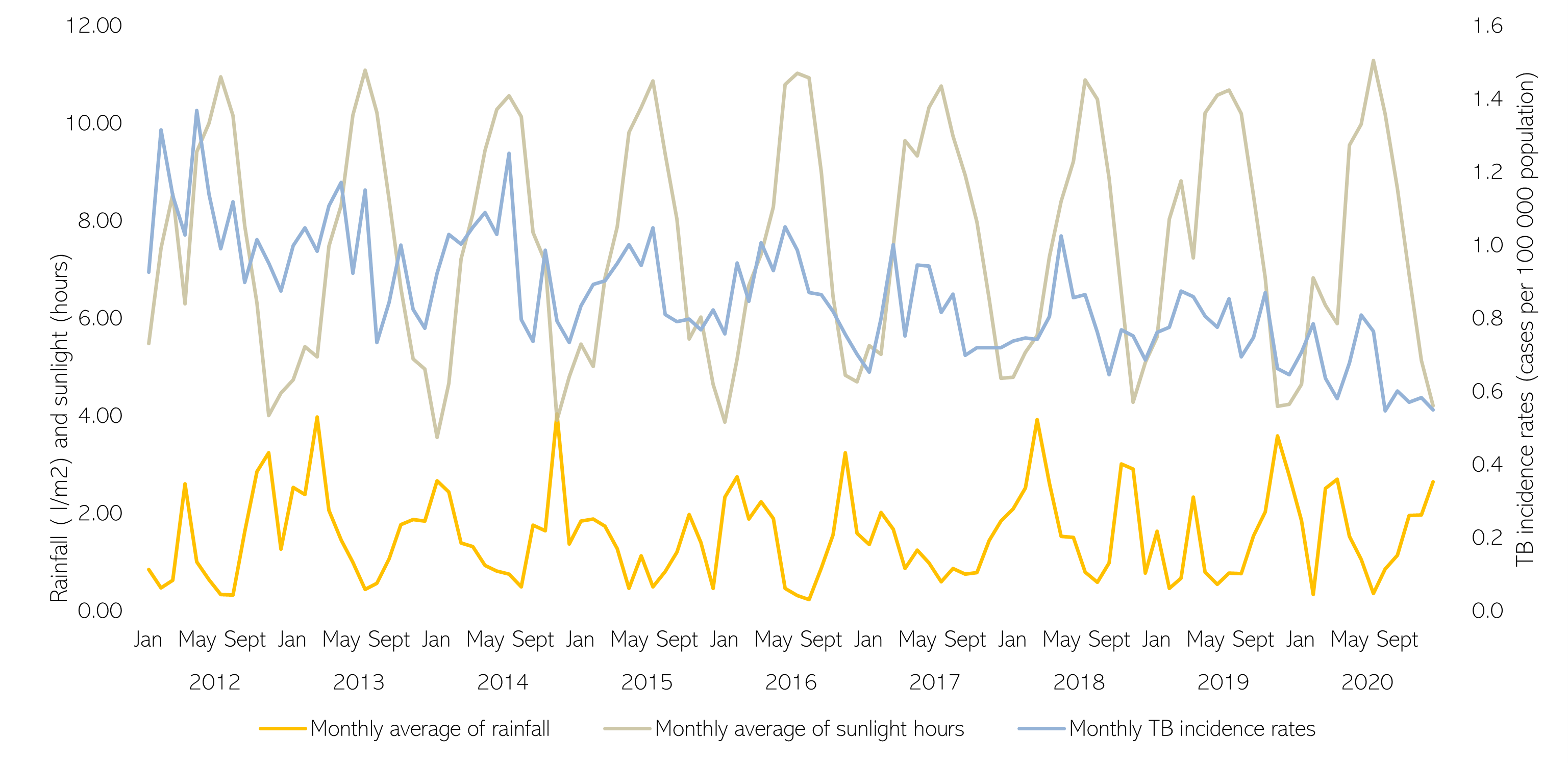

Supplement: Díez Galán et al. supplementary material [file S0950268824000499sup001.png]
